# Barriers & Facilitators of Innovation in Long-Term Care Homes During COVID-19: Insights from Brazilian experts

**DOI:** 10.1093/geroni/igaf122.3023

**Published:** 2025-12-31

**Authors:** Gilciney Rabello, Suzanne Santos, Gabriela Nacimben, Marisa Domingues, Meire Cachioni, Patrick Wachholz, Charlene Chu, Ruth Melo

**Affiliations:** Universidade de São Paulo, São Paulo, Sao Paulo, Brazil; Universidade de São Paulo, São Paulo, Sao Paulo, Brazil; Universidade de São Paulo, São Paulo, Sao Paulo, Brazil; Universidade de São Paulo, São Paulo, Sao Paulo, Brazil; Universidade de São Paulo, São Paulo, Sao Paulo, Brazil; Universidade Estadual Paulista, Botucatu, Sao Paulo, Brazil; University of Toronto, Toronto, Ontario, Canada; Universidade de São Paulo, São Paulo, Sao Paulo, Brazil

## Abstract

The COVID-19 pandemic profoundly disrupted global healthcare systems, with Long-Term Care Homes (LTCHs) facing unprecedented challenges that underscored the critical role of innovation in crisis response. This cross-sectional qualitative study, part of the CONNECTED consortium, examined insights from 12 Brazilian experts (researchers, LTCH managers, industry representatives, and heads of local/national LTCH organizations) to explore barriers and facilitators in implementing innovations during COVID-19. Data were collected through 12 semi-structured interviews (from March to October 2024) and analyzed using thematic analysis. The innovations implemented during COVID-19 were related to: 1) safety and preventive measures, 2) communication and social connections tools, 2) telehealth, 3) remote training programs for LTCH staff, and 4) national voluntary movement to support the LTCHs. Interviewees identified multiple systemic barriers to innovation implementation in LTCHs, including resource constraints (financial and human), digital illiteracy among residents, institutional resistance to change, inadequate staff training, regulatory hurdles, infrastructural limitations, and entrenched cultural stigma surrounding LTCH care. Conversely, experts highlighted key facilitators: targeted governmental and organizational funding, national-driven knowledge sharing, implementation of rapid communication channels, and cross-sector partnerships between public health authorities and LTCHs—all of which proved critical in mitigating pandemic-related disruptions. In Brazil, while the COVID-19 pandemic pressured catalyzed rapid adoption of vital innovations, from telehealth platforms to national solidarity initiatives, systemic barriers like resource scarcity, infrastructural gaps, and cultural stigma persisted as ingrained systematic issues. Therefore, addressing these systemic challenges is imperative to develop the resilience of the Brazilian long-term care sector and ensure preparedness for future health crises.

